# Efficiency based updating of evidence based surgical guidelines - Experiences from a multidisciplinary guideline upon Minimally Invasive Surgery

**DOI:** 10.52054/FVVO.15.3.088

**Published:** 2023-09-24

**Authors:** F.H.M.P. Tummers, S.F.P.J. Coppus, B.W. Lagerveld, A Demirkiran, E.S. van Schrojenstein Lantman, T.A. Brouwer, W.A. Draaisma, F.W. Jansen

**Affiliations:** Department of Gynecology, Leiden University Medical Center, 2333 ZA Leiden, The Netherlands; Department of Gynecology, Maxima Medical Center, 5631 BM Veldhoven/Eindhoven, The Netherlands; Department of Urology, OLVG, 1091 AC Amsterdam, The Netherlands; Department of Surgery, Red Cross Hospital, 1942 LE Beverwijk, The Netherlands; Department of Medical Technology and Medical Physics, Albert Schweitzer Hospital, 3318 AT Dordrecht, The Netherlands; Department of Anesthesiology, Medical Center Leeuwarden, 8934 AD Leeuwarden, The Netherlands; Department of Surgery, Jeroen Bosch Hospital, 5223 GZ Den Bosch, The Netherlands; Department of BioMechanical Engineering, Delft University of Technology, 2628 CD Delft, The Netherlands

**Keywords:** Guideline development, Guideline updating process, Minimally Invasive Surgery, Surgical Guidelines, New techniques

## Abstract

**Background:**

Updating evidence-based clinical practice guidelines is an onerous process and there is a call for more efficient determination of key questions that need updating. Especially for surgical techniques it is unclear if new evidence will result in substantial changes after wide implementation and if continuous updating is always necessary.

**Objectives:**

This study analyses the impact of updating a surgical guideline and proposes suggestions for optimising this process.

**Material and Methods:**

The Dutch Minimally Invasive Surgery guideline was developed in 2011 and updated in 2021. For both versions a multidisciplinary guideline working group (GDG) was created, that determined key questions. Changes in conclusions and recommendations were analysed by the GDG and statements for expected change of recommendations in the future were made.

**Results:**

15 key questions were formed, of which 12 were updates of the previous guideline. For only 27% of the updated key questions, the conclusions changed. In ten years, the body grew only marginally for most key questions and quality of the evidence did not improve substantially for almost all key questions. However, in this first update of the MIC guideline, many recommendations did change due to a more robust interpretation of the conclusions by the GDG. Based on analysis of this updating process, the GDG expects that only four out of 15 recommendations may change in the future.

**Conclusion:**

We propose an additional step at the end of guideline development and updating, where the necessity for updating in the future is determined for each key question by the GDG, using their valuable knowledge gained from developing or updating the guideline. For surgical guidelines, the authors suggest updating key issues if it includes a relatively newly introduced surgical- or adapted technique or a new patient group. Low quality or small body of evidence should not be a reason in itself for updating, as this mostly does not lead to new evidence-based conclusions. This new step is expected to result in a more efficient prioritising of key questions that need updating.

**What's new?:**

By adding one additional step at the end of the updating process, the future updating process could become more efficient.

## Introduction

Evidence-based clinical practice guidelines (CPG) include recommendations intended to optimise patient care. These recommendations are based on a systematic review of evidence and help to translate evidence into clinical practice (Grimshaw et al., 2004; Steinberg et al., 2011; Woolf et al., 1999; World Health Organization et al., 2014). The growing volume of CPGs is, however, overwhelming and at this moment the Dutch Guideline Database for hospital care consists of 450 guidelines (Federatie Medisch Specialisten, 2022).

Due to systemic reviewing of the extensive available literature, guideline development is a demanding and time-consuming process, while it should be both efficient and cost effective (McDonald et al., 2019). Because of rapidly changing and evolving clinical evidence, guidelines need to be updated to contain the most recent evidence and maintain validity (Shekelle, 2014; Shekelle et al., 2001). Although multiple handbooks are written on how to develop a guideline ( National Institute for Health and Care Excellence , 2012; Scottish Intercollegiate Guidelines Network, 2019), only little is available for updating a guideline (Martínez García et al., 2012; Vernooij et al., 2014). Also, the updating process takes a lot of resources, which was already acknowledged by Vernooij et al. (2014). They discussed several methods for this process, proposed a first step towards a pragmatic approach and developed a checklist for reporting it (Martínez García et al., 2012; Vernooij et al., 2017; Vernooij et al., 2014). Agbassi et al. (2014) created a pragmatic process to prioritize CPGs in the need for an update. Despite these suggestions, updating CPGs is not yet an efficient process.

In particular, guidelines on new (surgical) techniques and technologies are labelled with the need to be updated after implementation as it is expected that new data will become available regarding their clinical impact. However, for these guidelines, it is unlikely evidence will change substantially once techniques are widely implemented (Cuss et al., 2015).

The aim of our study is to pose suggestions for optimising the efficiency of this process based on experiences of a multidisciplinary surgical guideline updating process.

## Materials and Methods

For this manuscript, we used the Dutch Minimally Invasive Surgery (MIS) guideline to analyse the updating process. This guideline was first developed in 2011 (la Chapelle et al., 2012) and updated in 2021 (Nederlandse Vereniging voor Obstetrie en Gynaecologie, 2021). The full updated guideline is published online in Dutch. In the first version, a time frame for updating of 5 years was set, or in case new developments resulted in the necessity for revision. Despite this time frame, the eventual updating process started in 2019, 8 years after the publication of the guideline. This was mainly due to available resources, prioritising of other guidelines, and time constraints.

For both versions, a multidisciplinary guideline development group (GDG) was created to respectively develop and revise the guideline. One gynaecologist (FWJ) was member of both GDGs.

Both guidelines were developed with the Appraisal of Guidelines for Research and Evaluation (AGREE) II instrument (Brouwers et al., 2010). The GDG performed identification of key clinical issues. For all key clinical issues of the previous guideline, it was determined that if these were still a key issue now and if it was expected that updating these issues would lead to new or stronger evidence and recommendations. 11 issues were considered relevant for updating and 15 were not considered for updating. Additionally, 2 new key clinical issues were formed regarding topics that were not covered in the previous guideline. Table I shows all key questions of the 2021 guideline.

**Table I:** Key questions for the 2021 guideline.

KQ nr.	Key question	New or update
1	Which entry technique is preferred for adults with an indication for laparoscopy: open or closed?	Update
2	Which entry technique is preferred in adults with an indication for laparoscopy: an alternative entrance technique versus open or closed?	Update
3	Which entry technique is preferred in pregnant women that need laparoscopic surgery?	Update
4	Which entry technique is preferred for adults with BMI lower than 18?	Update
5	Which entry technique is preferred for adults with BMI above 30?	Update
6	Which entry technique and which entry location is preferred for re-laparoscopy in adults?	Update
7	Which electrosurgical techniques can be used safely based on complications?	New
8	Do the fascia have to be closed with trocar site openings of ≤ 10mm and/or > 10 mm?	Update
9	What is the most suitable method for closing the fascia?	Update
10	Which intra-abdominal pressure in a CO2 pneumoperitoneum is preferred in adults?	Update
11	At what intra-abdominal pressure can the first trocar be inserted in adults?	Update
12	Does the use of a deep neuromuscular block provide surgical benefits for low pressure pneumoperitoneum in adults?	New
13	Should the trocar site be infiltrated with anesthetic in adults? If so, should this site be infiltrated with a short-acting or long-acting anesthetic? And at what dosage and time?	Update

For the 2011 guideline, the methodological quality of the studies was assessed using the Grading System from the Dutch Institute for Healthcare Improvement (CBO), which consisted of levels A-D for study quality and conclusion quality level A-D. For the 2021 guideline, the Grading Recommendations Assessment, Development and Evaluation (GRADE) system was used. GRADE distinguishes four grades for the quality of scientific evidence: high, moderate, low, and very low. (Hultcrantz et al., 2017; Schünemann et al., 2013).

The key questions were analysed for both versions of the guideline. Evidence level, recommendations, and changes in recommendations were noted. Additionally, after finishing the guideline, the 2021 GDG members, also being the authors of this manuscript, considered all changes in evidence and recommendations and prepared a statement for each recommendation if it is expected that future updating would result in new recommendations, which resulted in this manuscript.

## Results

Table II shows the changes in recommendations compared to the previous guideline, including the quality of evidence and the reason for the change. The 13 key questions resulted in 15 recommendations, based on 15 evidence-based conclusions. 12 recommendations were updates from the previous guideline, and 3 recommendations were new.

**Table II:** Recommendation 2011 compared to 2021. *Evidence level is based on crucial outcome.

KQ nr.	2011	Evidence level*	2021	Evidence level*	Change of evidence-based conclusion?	Change of recommendation? (including reason)	Expected new evidence in future that changes recommendation OR relevant new evidence
1	There is no general recommendation regarding the safest type of entrance technique. Special- ists can best stick to the technique they have learned and are familiar with. The opinion is that experi- ence with a specific entrance technique limits the risks. The GDG does not recommend the use of direct trocar entry, as a great deal of experi- ence is required to be able to apply this technique safely.	Level 1, A1	Choose the technique that the operator has learned and is familiar with, so that the risks are minimized through experience. Be aware of the different entrance techniques and entrance locations. It is important that the operator has the ability to switch techniques if necessary.	Very low	No	Open vs closed not changed. Within closed: direct trocar is a considered a technique within the closed entry group. The direct trocar technique is now considered a safe technique. Added: know other techniques, so you can switch if necessary.	No. Evidence did not change in last years and all techniques are widely used. It is not expected to be a key clinical issue anymore in the future.
2	It is recommended to use direct vision entry only when the pneu- moperitoneum has been created before- hand (with the Veress needle).	Level 3, C	The direct vision entry appears to be a safe alternative to the open or closed entry technique, even without pneumoperitoneum prior to insertion.	Very low	No, but larger body of evidence	Yes. This technique is also allowed without prior pneumoperito- neum as more studies using this technique are available. The evidence conclusion however did not change.	No. Although no direct comparison is available for prior pneumoperi- toneum vs no prior pneumoperito- neum, both tech- niques have been described without additional risks. Next to that, direct trocar insertion is now also considered a safe technique. It is not expected to be a key clinical issue anymore in the future.
3	In a pregnant patient who has to undergo a laparoscopic procedure, an open entrance technique is preferred.	Level 4, D	In a pregnant patient for whom laparoscopic surgery is indicated, choose the entrance technique with which the surgeon is familiar. Adjust the entrance location based on the height of the uterus. Palmer’s point could be considered as an alternative location for the entrance.	No GRADE	No, still little evidence available	Yes. All techniques are allowed, as all techniques are described with safe use.	No. Little evidence available, but it not increased in previous 10 years. In clinical practice all techniques are safely used.
4	In patients with underweight (BMI < 18 kg/m2) and children, the open (Hasson) technique or entrance via Palmer’s point is recommended.	Level 4, D	When using a closed entrance technique in adults with a BMI <18, be aware of the short distance from the navel to the underlying aorta. In these patients, consider alternative techniques such as Palmer’s point entry or the open entry technique.	No GRADE	No, still little evidence available.	Yes, based on same evidence conclusions, the GDG expert opinion now considers all techniques to be performed safely, but be aware of the risk of the adjacent aorta and consider alternative entry location of technique.	No. Still little evidence available, but it has not increased in previous 10 years
5	In patients with morbid obesity (BMI > 40 kg/m2), the closed entrance technique or entrance at Palmer’s point is recommended. When using the closed entry technique with Veress needle, care should be taken to place the incision at the base of the navel and to insert the needle vertically into the peritoneum.	Level 4, D	Be aware that entry into the abdominal cavity of a patient with a BMI>30 may be more difficult than a patient with a BMI 18-30 and that the internal anatomy may be displaced from the abdominal wall. For patients with a BMI>30, choose the technique that the operator has learned and is familiar with, so that the risks are limited through experience.	Very low	No, but larger body of evidence.	Yes. Previous recommendation was based on GDG expert opinion. All techniques are described in literature and used in clinical practice. Direction of Veress needle is no longer advised as evidence was inconsistent.	No. Little additional evidence and all techniques are safely used in clinical practice.
6	It is not recommended to perform a closed release in the area where adhesions are expected. In addition to the open technique, a closed technique through Palmer’s point may be recommended as an alternative site for insertion of the Veress needle and/or head trocar	Level 3, C	Be aware of possible adhesions after a previous laparoscopy. If you suspect adhesions and want to use the closed entrance technique: Consider alternative entrance location such as the closed entrance technique through Palmer’s point, or alternative techniques such as the open entrance technique or the under-view technique. Choose the alternative with which experience is available.	Very low	No, but in the previous guideline there was almost no evidence, now the body of evidence is larger.	Yes. It is a more open recommendation. You are allowed to perform the closed procedure. However, you should be aware of the potential adhesions and consider alternative options.	Potentially. In the coming years the patient group with repeat laparoscopy will grow, with po- tentially more research. However, the chance that new RCTs will come available which result in new recommendations (i.e., not allowed to open through the previous location) is small.
7	X		All electrosurgical techniques can be used in a safe manner if the working mechanism and risk of complications is known with all users.	Very low	X	New recommendation	No, all techniques are widely used and considered safe. It is not expected to be a key clinical issue anymore in the future.
8	It is recommended to close the fascia of trocar insertion holes with a size of >10 mm. It may also be worth considering closing smaller ports, for example in the case of risk factors.	Level 3, B	Consider not closing the fascia of trocar insertion openings with a size of ≤ 10 mm and closing the fascia from trocar insertion openings > 10 mm.	Very low	Yes	It is not recommended anymore to close smaller openings as evidence suggests no difference for herniations.	No, still little evidence is available and not expected to expand, resulting in new recommendations.
9	There are no specific recommendations for closing the fascia. When suturing the fascia, transmural suturing should be considered. This may reduce the risk of subfascial hernias	Level 4, D	There are no specific recommendations for the method by which the fascia should be closed.	No GRADE	No	Yes. Transmural closing is not recommended anymore as no sufficient evidence supported this anymore.	No, no new evidence is expected. It is not expected to be a key clinical issue anymore in the future.
10	An absolute upper limit of intra-abdominal pressure in a CO_2_ pneumoperitoneum cannot be indicated. In view of the cardiovascular and pulmonary side effects, it is preferable to operate with the lowest possible intra- abdominal pressure with adequate exposure of the surgical site.	Level 1, A1 and level 2, A2	Preferably use a standard intra-abdominal pressure (12-15 mmHg) Consider deviating from this in consultation with the anaesthesiologist if this is desirable due to the patient’s comorbidity. In ASA 3-4 patients, depending on the patient’s comorbidity, consult the anaesthetist about the expected pressure that the patient can accept.	Very low	Yes	Yes. Pneumoperitoneum is advised to be normal instead of as low as possible due to large body of new evidence.	Yes. NMB is progressing, so potentially new evidence becomes available, powered for complications, showing better difference between low and normal pressure.
11	The hyperdistention technique may only be used for a short time during the entrance.	Level 3, B	Short-term hyper- extension pressure before introduction of the trocars does not appear to be harmful for ASA 1-2 patients.	Very low	No	No	No, no new evidence is expected.
12	Prior to closed entrance to the main trocar, intra- abdominal pressure should be at least 12 - 16 mm Hg, depending on patient characteristics. Hyper insufflation up to 20-25 mm Hg may be used for a short period of time in a select group of patients	Level 3, C	Preferably perform the entrance at a standard intra-abdominal pressure (12-15 mmHg).	Very low	No	Yes. Normal pressure is advised, higher pressure is not necessary.	No, no new evidence is expected.
13	It is preferable to infiltrate the insertion openings at laparoscopy with a long-acting local anaesthetic, such as bupivacaine, in order to reduce early postoperative pain.	Level 1, A1	For laparoscopy, consider infiltrating the insertion site with a long-acting anaesthetic to reduce postoperative pain. The GDG cannot make a recommendation on the dosage and timing of the infiltration of the anaesthetic.	No GRADE	No	No	Potentially. For dosing and timing no sufficient evidence is available now. This might improve in the coming years, but it hasn’t in the last 10 years.
14	X		Consider choosing deep NMB to create better surgical conditions if low intra- abdominal pressure is chosen.	Low	X	New recommendation	Yes, NMB is a new technique, therefore more evidence is expected in the coming years.
15	X		Monitor muscle relaxation perioperatively and regulate the depth of the block based on these measurements.	No GRADE	X	New recommendation	No. This recommendation is related to the practical use of NMB during surgery and not expected to change in the future.

For 2 key questions, the conclusions did not change, and the recommendations also stayed the same. For 6 questions, the evidence- based conclusions did not change, but the recommendations were changed. This was mostly because of more robust interpretation of the evidence by the GDG or more widely experience with the technique. In the 2011 guideline, little evidence was available, and the techniques were relatively new. Therefore, many recommendations were authority based to give the much-needed guidance at that moment. However, with now more experience with all techniques, the GDG changed the recommendations in accordance with the evidence-based conclusions. For 3 questions the conclusions did not change, however due to a larger body of evidence, the recommendation changed. For 2 questions the conclusions changed and therefore the recommendations.

Even with a growing body of evidence, almost all evidence was graded very low GRADE, despite only including randomized controlled trials (RCT) and systematic reviews (SR). The evidence was mostly downgraded due to imprecision and risk of bias.

For 11 recommendations, the GDG expects that the future updating would not result in a change of the recommendation or that this topic is not a key clinical issue anymore in the future. This is suggested as the techniques are widely used and all considered safe, or because no new evidence is expected in the future as this has not appeared in the last 10 years. For four recommendations the GDG expects or believes it possible that new evidence will be available which may change recommendations. For example, because the described patient group is growing or because it describes a new technique.

## Discussion

Our results show that after 10 years, most evidence- based conclusions have not changed. For most key questions, the body of evidence either stayed the same or grew without changing the conclusions. However, for some, the body grew and resulted in clinically relevant different conclusions. The quality of evidence was still low or very low for most issues, despite exclusively including RCTs and SRs and some growth in the body of evidence. For some questions, the evidence-based conclusions mostly did not change, but recommendations did. This was largely due to the more robust interpretation by the GDG and wider implementation of the technique for special patient groups and alternative techniques.

The efficiency of a surgical guideline updating process is cumbersome. In future updating, we should ask ourselves if this could be performed more efficiently and what could aid to this goal.

Albeit it is important that guidelines reflect the currently available evidence; estimating when and if an update of recommendations will result in changes is difficult.

Vernooij et al. (2014) already recognized the resourceful updating process and lack of guidance and made suggestions to optimise this. One of their main suggestions is to first search for evidence, then assess if there is a need for updating, and only update those questions with a high probability of change. Nonetheless, these first two steps are still time-consuming, as many references need to be screened with this method (Martínez García et al., 2012). Restricted searches could reduce this, but pose a risk of missing important data (Martínez García et al., 2012). The research group of Martinez Garcia recently developed a tool to optimise the updating process, which shows great potential. However, in these mentioned proposals still all research questions need to be analysed (Martínez García et al., 2017; Sanabria et al., 2021; Sanabria et al., 2020). The use of resources could be optimised if there would be no need to check the evidence for all existing key questions.

As many authors recognise the limited resources available for updating processes, the need for an optimal selection process for partial updating is high (Becker et al., 2018; Goossen et al., 2022; Sanabria et al., 2021). Agbassi et al. (2014) suggested a prioritising system for CPGs by a methodologist with an option not to update if the existing evidence is solid. This could be based on already available high-quality evidence, an adequate body of evidence or if no additional evidence will be forthcoming because it is no longer a key issue. However, their results showed that none of the 103 screened CPGs were actually labelled as endorsed (Agbassi et al., 2014). Sanabria et al. (2021) add to this by mentioning that new GDG members might cause disagreement regarding the need for updating due to the lack of previous experience in the clinical issue topic. Our key issue analysis already considered part of the old questions not relevant for updating. Yet, for the remaining questions that were updated, only a small part of the evidence-based conclusions changed, whereas the GDG expected differently.

The above-mentioned highlights that it can be difficult for the new GDG to determine which key issues do not need updating. In our opinion, the current GDG could be of added value in this prioritising step. This GDG has mastered the available evidence and has insight in the need and value of new evidence that can change the conclusions. Additionally, many prioritising systems suggest the first steps of prioritising performed by epidemiologists. Although we encourage epidemiologic support during updating processes, we do believe that clinicians should be leading prioritising. As the time span between development and updating or between updating processes is long, the new GDG composition has often changed, inherently the valuable knowledge of the old GDG group is lost.

Key issues are often considered relevant for updating if the previous conclusions were based on low quality or a low body of evidence. Studies have shown that low-quality evidence in CPGs is a frequent problem in various fields of medicine, including surgical ones (Brito et al., 2013; Cooper et al., 2015; Duarte-García et al., 2018; Geoffrion and Larouche, 2021; Lee and Vielemeyer, 2011; Murad et al., 2011; Pandis et al., 2015; Rowe et al., 2012; Sardar et al., 2019; Tricoci et al., 2009; Yong et al., 2019). However, low-quality evidence might in some cases still result in strong recommendations (Schünemann et al., 2013), and weak recommendations can give significant clinical guidance in the absence of better options (Guyatt, 2018; Schoemaker et al., 2018). Our results show that the quality and body of evidence have not changed much in 10 years. Furthermore, it is doubtful if the quality will improve in the coming decade, or that the conclusion would change despite the growth of evidence. In line with our research, Lyratzopoulos et al. (2012) showed that updating because of inadequate evidence only led to changes in about half of the recommendations. In 2014 it was already suggested that we may have to accept the evidence for laparoscopic entry techniques as being as good as it gets (Cuss et al., 2015).

To optimise efficiency of selection of key issues for updating, we propose a new crucial step in surgical guideline development and updating. At the end of the process, after formulating the recommendations, the GDG states if it is likely that recommendations will change in the future, including the expectation if an issue will still be a key issue in the future. This statement is based on the already available evidence, the changes in evidence in the last years, and implementation of the techniques. We have implemented this suggestion for the updated guideline (Table II). Our results showed that 11 out of 15 questions presumably do not need updating in the future, as the chances of relevant change in recommendations are considered to be very low. Especially for techniques that are already widely implemented, the GDG should discuss if they need updating in the future, as the issues might not be considered clinically relevant anymore. For example, according to our GDG, the key question regarding a preferred entry technique (open or closed) is not relevant anymore as many agree that all options are safe and long-term evidence shows equal risk of complications. Low- quality evidence or a small body of evidence is no reason in itself to update it in the future. Key issues that need updating include relatively new techniques (e.g., neuromuscular blockage (NMB)), new patient groups (e.g., re-laparoscopy), or new techniques or innovations added to the surgical palette. These statements are a handle for the future GDG and should not be seen as a definitive discretion. The future GDG can reassess the given advice if this is considered relevant in the light of new clinical issues or new evidence. Our proposed step could provide additional information when using newly developed priority tools, or it could prevent the key issues from entering the prioritising process if updating is not considered necessary.

For our updated MIS guideline, this first extended updating process was valuable. Although only a small amount of evidence-based conclusions changed, multiple recommendations changed. During the development of the first MIS guideline, there was a high need for clinical guidance using these new techniques. Although evidence-based conclusions showed no superiority of a specific technique, the GDG offered authority-based guidance in the recommendation as the conclusions was based on limited evidence and experience with the techniques varied. However, although many conclusions did not change substantially in the updated guideline, the GDG now felt reassured to make recommendations according to the conclusions, as the experience with all techniques has grown substantially and sometimes the body of evidence also grew. These experiences strengthen our recommendation to suggest at least one future update for recommendations regarding new techniques. However, after wide implementation and the conclusion that little new evidence has come available, there is not always the need to keep updating all questions. In case of our MIS guideline, only a small number of questions need updating in a future update and resources could be spent on other clinical questions.

Although we describe experiences from a surgical guideline process, we do believe our suggestions are generalisable to other medical fields. The need for prioritisation is not limited to surgical guidelines, and developed prioritizing steps also are suggested to implement widely (Martínez García et al., 2017; Sanabria et al., 2020; Vernooij et al., 2017; Vernooij et al., 2014). Therefore, we believe our suggested step could be used in guidelines throughout the whole medical field. However, for every guideline it is important to be critical during the updating process. It could be important for guidelines including for example pharmaceutical treatments to keep updating all key questions. However, for all medical fields, our suggested step encourages the GDG to critically think about which key questions probably need updating in the future, using the valuable knowledge of the current GDG, which could result in a more efficient process.

In a world with limitless resources, full updating processes are justifiable, however, this is not contemporary reality. In the present time we need to be more efficient with resources and we should focus on efficient ways to perform the best evidence- based care possible. Our proposed step could aid in this need. Surely, the added value of our suggested step may vary per guideline. For all guidelines, our proposed step, costing little effort, is expected to result in a more efficient key issue analysis with the chance of a more selective group of questions with the need for an update.

## Conclusion

Updating a guideline is a resourceful process and more efficiency is needed. It is difficult to determine which key issues are relevant to update for a new GDG at the beginning of the process. We propose an additional step by the GDG at the end of the development and updating process of a guideline, where statements are made regarding the expectations that future updating would result in changes of recommendations. The necessity for future updating is determined for all single key questions. In our opinion, one should update key questions if it is a relatively new technique, a new patient group or if a new technique or innovation was added to the surgical palette. However, low quality or a small body of evidence should not be a reason in itself for updating a guideline, as this does not necessarily lead to new evidence-based conclusions.
